# Evaluation of a Latex Agglutination Assay for the Identification of *Burkholderia pseudomallei* and *Burkholderia mallei*

**DOI:** 10.4269/ajtmh.14-0025

**Published:** 2014-06-04

**Authors:** Brea D. Duval, Mindy G. Elrod, Jay E. Gee, Narisara Chantratita, Sarunporn Tandhavanant, Direk Limmathurotsakul, Alex R. Hoffmaster

**Affiliations:** Bacterial Special Pathogens Branch, Centers for Disease Control and Prevention, Atlanta, Georgia; Department of Microbiology and Immunology, Faculty of Tropical Medicine, Mahidol University, Bangkok, Thailand; Mahidol-Oxford Tropical Medicine Research Unit, Faculty of Tropical Medicine, Mahidol University, Bangkok, Thailand; Department of Tropical Hygiene, Faculty of Tropical Medicine, Mahidol University, Bangkok, Thailand

## Abstract

Cases of melioidosis and glanders are rare in the United States, but the etiologic agents of each disease (*Burkholderia pseudomallei* and *Burkholderia mallei*, respectively) are classified as Tier 1 select agents because of concerns about their potential use as bioterrorism agents. A rapid, highly sensitive, and portable assay for clinical laboratories and field use is required. Our laboratory has further evaluated a latex agglutination assay for its ability to identify *B. pseudomallei* and *B. mallei* isolates. This assay uses a monoclonal antibody that specifically recognizes the capsular polysaccharide produced by *B. pseudomallei* and *B. mallei*, but is absent in closely related *Burkholderia* species. A total of 110 *B. pseudomallei* and *B. mallei* were tested, and 36 closely related *Burkholderia* species. The latex agglutination assay was positive for 109 of 110 (99.1% sensitivity) *B. pseudomallei* and *B. mallei* isolates tested.

The Gram-negative bacteria *Burkholderia pseudomallei* and *Burkholderia mallei* are the etiologic agents of melioidosis and glanders, respectively. Melioidosis typically causes disease in humans and is endemic to Southeast Asia and northern Australia, whereas glanders is a disease most commonly seen in horses, mules, and donkeys in the Middle East, Africa, and India. Both bacteria are of concern because of their potential use as bioterrorism agents. The rarity of both diseases in the United States and other countries where the diseases are not endemic could delay proper diagnosis by physicians and laboratory staff during a bioterrorism event caused by responders' unfamiliarity with the diseases. Diagnostic confirmation of both diseases relies on microbiological culture. However, *B. pseudomallei* is commonly dismissed as a culture contaminant, and along with *B. mallei* may be misidentified by standard identification methods including API 20NE and other automated bacterial identification systems. Therefore, rapid diagnostic tools for bacterial identification are essential to provide an effective response by public health authorities in the event of a bioterrorism incident. The goal of this study was to evaluate a rapid assay for the identification of *B. pseudomallei* and *B. mallei.*

Latex agglutination assays have been used successfully in Southeast Asia and northern Australia to identify *B. pseudomallei* isolates and closely related species.^1^ Assays such as these are based on the use of monoclonal antibodies (MAbs) that recognize an exopolysaccharide present on the cell surface of *B. pseudomallei* and *B. mallei*.^2^–^4^ Nonetheless, these assays are normally evaluated with limited strains isolated from endemic areas, and its use for strains isolated from all other countries has not been adequately evaluated.^2^–^4^

Our laboratory has evaluated a rapid latex agglutination assay developed by Mahidol University (Bangkok, Thailand) using an inclusivity panel of 110 geographically and genetically diverse *B. pseudomallei* and *B. mallei* isolates, stored at The Centers for Disease Control and Prevention (CDC), Atlanta, GA. We also evaluated the assay with an exclusivity panel of 36 closely related *Burkholderia* species, which included agents that have not been previously tested by this or similar antigen detection assays. We focused on the closest phylogenetic relatives of *B. pseudomallei* including other *Burkholderia* species that have been associated with human disease such as *Burkholderia oklahomensis* and *Burkholderia gladioli*. *Burkholderia oklahomensis* has been reported to cause infections associated with deep tissue wounds,^5^,^6^ whereas *B. gladioli* can cause a range of diseases from fatal foodborne illness,^7^ to sepsis in newborns,^8^ and lung infections in patients with cystic fibrosis.^9^ This latex agglutination assay could be valuable in correctly identifying select agents and excluding closely related *Burkholderia* species that cause similar disease in humans.

The antibody-latex suspension based on the 4B11 monoclonal antibody was prepared by Mahidol University as previously described.^2^–^4^ The assay was performed also as previously described with slight modification.^1^ Briefly, isolates were subcultured twice on trypticase soy agar (TSA) containing 5% sheep's blood and incubated for 18–24 hours at 37°C. Single colonies were picked and added to 10 μL of the latex suspension on a ringed glass microscope slide. The glass slide containing the latex suspension with the suspended colony was subjected to gentle rocking for 2 minutes after which time the reaction was recorded as either positive (agglutination) or negative (no agglutination) (Figure 1). *Burkholderia pseudomallei* K96243 was used as the positive control in all experiments and *Burkholderia thailandensis* E264 (American Type Culture Collection [ATCC] type strain 700388) was used as the negative control each time isolates were tested, and all tests were performed in triplicate.

**Figure 1.**
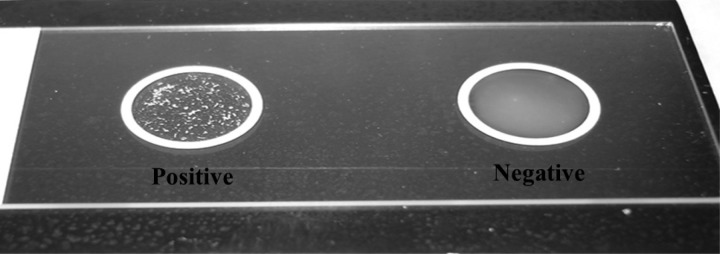
*Burkholderia pseudomallei* and *Burkholderia thailandensis* positive and negative reactions after incubation with the latex agglutination reagent.

Under our assay conditions, the latex agglutination test was positive on 109 of 110 (99.1% sensitivity) isolates tested on the inclusivity panel. This number included a total of 77 *B. pseudomallei* isolates, of which 76 (98.7% sensitivity) were positive and 33 *B. mallei* isolates of which all were positive (100% sensitivity) (Table 1). The *B. pseudomallei* isolate that tested negative in our assay, CDC2721686 (MSHR1655), was isolated from a patient with a chronic *B. pseudomallei* infection after being first diagnosed with melioidosis in 2000.^10^ This rare *B. pseudomallei* isolate was from the patient in an ongoing study consisting of 815 patients that were culture-positive for melioidosis in Darwin, Australia. Since 1989, this patient is the only survivor from this study to remain chronically colonized by *B. pseudomallei*. CDC2721686 (MSHR1655) was isolated 37 months after the initial melioidosis diagnosis and has undergone major genome-wide rearrangements resulting in a loss of function in many genes that are important in pathogenesis. Of particular interest to this study is the loss of function of *wcbR*, which encodes an essential fatty acid synthase required in capsular polysaccharide synthesis.^11^ We believe this would explain the inability of the latex agglutination assay to correctly identify this isolate. In addition to testing negative in our assay, when subjected to standard biochemical tests for the identification of *B. pseudomallei*, the isolate was non-motile, but otherwise normal under our assay conditions. When the latex agglutination assay was tested against an exclusivity panel of closely related *Burkholderia* species, 35 of 36 (97.2% specificity) yielded negative results (Table 2). The closely related *Burkholderia* that tested positive in our assay is a rare variant of *B. thailandensis* (CDC3015869, TX DOH) that has been previously described as containing *B. pseudomallei* capsule genes.^12^

Rapid diagnostic assays, such as the one we have evaluated, would have the most impact in clinical laboratories. This would allow for early identification of suspect isolates and thus on-site diagnosis instead of needing to submit samples to regional laboratories that would delay results. This assay does have several advantages over the current reference level testing. This assay is simple, does not require extra equipment, and can easily be performed. However, the extent to which this assay or similar antigen detection assays can be used on patient samples is yet to be determined.

## Figures and Tables

**Table 1:** *Burkholderia pseudomallei* and *Burkholderia mallei* inclusivity panel

Species	Strain identifier	Location of origin	Result
*Burkholderia pseudomallei*	CDC2721620	France	Positive
*Burkholderia pseudomallei*	CDC2721628	Madagascar	Positive
*Burkholderia pseudomallei*	CDC2721639	Kenya	Positive
*Burkholderia pseudomallei*	CDC0022138	Thailand	Positive
*Burkholderia pseudomallei*	Bp92; CDC2721623	Australia	Positive
*Burkholderia pseudomallei*	Human 88; PHLS 45	Thailand	Positive
*Burkholderia pseudomallei*	Bp104; CDC2721624	Australia	Positive
*Burkholderia pseudomallei*	CDC2721635; PHLS 36	Singapore	Positive
*Burkholderia pseudomallei*	Bp73; Ln31348	Malaysia	Positive
*Burkholderia pseudomallei*	PHLS 208	Ecuador	Positive
*Burkholderia pseudomallei*	CDC2721102; F5013	Georgia	Positive
*Burkholderia pseudomallei*	BpG9709; CDC0032026	India	Positive
*Burkholderia pseudomallei*	PHLS 19; CDC2721625	Singapore	Positive
*Burkholderia pseudomallei*	CDC2721676	USA	Positive
*Burkholderia pseudomallei*	Bp2889; SID2889	Bangladesh	Positive
*Burkholderia pseudomallei*	CDC2721630; 7605	France	Positive
*Burkholderia pseudomallei*	Bp68; CDC2721641	Fiji	Positive
*Burkholderia pseudomallei*	PHLS 17; CDC2721619	Indonesia	Positive
*Burkholderia pseudomallei*	PHLS 38	Singapore	Positive
*Burkholderia pseudomallei*	1106a; CDC0022030	Thailand	Positive
*Burkholderia pseudomallei*	Bp53; CDC2721633	Thailand	Positive
*Burkholderia pseudomallei*	Bp24; CDC2721620	France	Positive
*Burkholderia pseudomallei*	BpG9313; CDC0032029	USA	Positive
*Burkholderia pseudomallei*	CDC2721162	Australia	Positive
*Burkholderia pseudomallei*	CDC2721114; G6715	USA (Ohio)	Positive
*Burkholderia pseudomallei*	CDC2721626	Thailand	Positive
*Burkholderia pseudomallei*	CDC0032028	USA (Ohio)	Positive
*Burkholderia pseudomallei*	CDC721096; 81A442	USA (New York)	Positive
*Burkholderia pseudomallei*	CDC0032024	Puerto Rico	Positive
*Burkholderia pseudomallei*	Thai NE Human 99	Thailand	Positive
*Burkholderia pseudomallei*	CDC1029240	USA (Oregon)	Positive
*Burkholderia pseudomallei*	CDC2721617	Australia	Positive
*Burkholderia pseudomallei*	Bp14; CDC2721618	Philippines	Positive
*Burkholderia pseudomallei*	BpH1442; CDC0032025	USA (Delaware)	Positive
*Burkholderia pseudomallei*	MSHR640;CDC8724880	Australia	Positive
*Burkholderia pseudomallei*	465a; CDC8724601	Australia	Positive
*Burkholderia pseudomallei*	MSHR99; CDC8724881	Australia	Positive
*Burkholderia pseudomallei*	CDC1756207	Australia	Positive
*Burkholderia pseudomallei*	CDC8724890	Australia	Positive
*Burkholderia pseudomallei*	#711; CDC2721675	USA (Washington)	Positive
*Burkholderia pseudomallei*	CDC2734678; 620	Thailand	Positive
*Burkholderia pseudomallei*	CDC8724908	Australia	Positive
*Burkholderia pseudomallei*	CDC8724883	Australia	Positive
*Burkholderia pseudomallei*	CDC2734694; PM40	Thailand	Positive
*Burkholderia pseudomallei*	PM26; CDC2734683	Thailand	Positive
*Burkholderia pseudomallei*	PHLS 75	Malaysia	Positive
*Burkholderia pseudomallei*	CDC8724901	Australia	Positive
*Burkholderia pseudomallei*	PM115; CDC2734709	Thailand	Positive
*Burkholderia pseudomallei*	CDC2721825	Thailand	Positive
*Burkholderia pseudomallei*	Bp40	Singapore	Positive
*Burkholderia pseudomallei*	CDC8724894	Australia	Positive
*Burkholderia pseudomallei*	CDC2734661; SA923	Thailand	Positive
*Burkholderia pseudomallei*	PHLS 79	Malaysia	Positive
*Burkholderia pseudomallei*	BpH1689; CDC0032024	USA (Florida)	Positive
*Burkholderia pseudomallei*	CDC2721184	Ecuador	Positive
*Burkholderia pseudomallei*	CDC2721634	Thailand	Positive
*Burkholderia pseudomallei*	CDC1756205	Australia	Positive
*Burkholderia pseudomallei*	CDC8724905	Australia	Positive
*Burkholderia pseudomallei*	CDC0022203	Thailand	Positive
*Burkholderia pseudomallei*	CDC2721637	Pakistan	Positive
*Burkholderia pseudomallei*	CDC8724896	Thailand	Positive
*Burkholderia pseudomallei*	CDC8724889	Australia	Positive
*Burkholderia pseudomallei*	CDC8724898	Australia	Positive
*Burkholderia pseudomallei*	CDC2721686	Australia	Negative
*Burkholderia pseudomallei*	CDC8724899	Thailand	Positive
*Burkholderia pseudomallei*	CDC8724882	Australia	Positive
*Burkholderia pseudomallei*	CDC8724900	Australia	Positive
*Burkholderia pseudomallei*	CDC8724892	Australia	Positive
*Burkholderia pseudomallei*	CDC8724893	Australia	Positive
*Burkholderia pseudomallei*	CDC2721761	Vietnam	Positive
*Burkholderia pseudomallei*	CDC8724885	USA	Positive
*Burkholderia pseudomallei*	CDC0022358	Thailand	Positive
*Burkholderia pseudomallei*	CDC8724877	Australia	Positive
*Burkholderia pseudomallei*	CDC1756206	Australia	Positive
*Burkholderia pseudomallei*	CDC8724895	Australia	Positive
*Burkholderia pseudomallei*	CDC8724903	Australia	Positive
*Burkholderia pseudomallei*	CDC8724878	Australia	Positive
*Burkholderia mallei*	CDC2721277	China	Positive
*Burkholderia mallei*	CDC2734821	China	Positive
*Burkholderia mallei*	CDC2721278	USA (New Mexico)	Positive
*Burkholderia mallei*	CDC0031066	India	Positive
*Burkholderia mallei*	CDC2734315	Turkey	Positive
*Burkholderia mallei*	CDC0031065	Turkey	Positive
*Burkholderia mallei*	CDC2734302	Turkey	Positive
*Burkholderia mallei*	CDC2734301	Turkey	Positive
*Burkholderia mallei*	CDC0031304	USA (Maryland)	Positive
*Burkholderia mallei*	CDC2721273	Burma	Positive
*Burkholderia mallei*	KC 235; CDC2721274	USA (Maryland)	Positive
*Burkholderia mallei*	KC0248; CDC4017733	USA	Positive
*Burkholderia mallei*	CDC2721279	USA (New York)	Positive
*Burkholderia mallei*	CDC2721280	Iran	Positive
*Burkholderia mallei*	CDC8724847	Unknown	Positive
*Burkholderia mallei*	CDC2734305	India	Positive
*Burkholderia mallei*	CDC2734303; GB10	India	Positive
*Burkholderia mallei*	CDC8724837	Turkey	Positive
*Burkholderia mallei*	CDC8724838	Turkey	Positive
*Burkholderia mallei*	CDC8724839	Turkey	Positive
*Burkholderia mallei*	CDC8724841	Turkey	Positive
*Burkholderia mallei*	CDC2734300	Turkey	Positive
*Burkholderia mallei*	CDC2734301	Turkey	Positive
*Burkholderia mallei*	CDC2734317	India	Positive
*Burkholderia mallei*	CDC2721275	China	Positive
*Burkholderia mallei*	CDC2734299	Hungary	Positive
*Burkholderia mallei*	CDC2734311	England	Positive
*Burkholderia mallei*	CDC0031063	Hungary	Positive
*Burkholderia mallei*	CDC0031064	India	Positive
*Burkholderia mallei*	CDC2721276	USA	Positive
*Burkholderia mallei*	CDC2721648	Burma	Positive
*Burkholderia mallei*	CDC2734312	Turkey	Positive
*Burkholderia mallei*	CDC2721280	Iran	Positive

**Table 2:** *Burkholderia* exclusivity panel

Species	Strain identifier	Location of origin	Result
*Burkholderia thailandensis*	CDC3015869	USA (Texas)	Positive
*Burkholderia thailandensis*	CDC2721621	France	Negative
*Burkholderia thailandensis*	CDC2721627	Thailand	Negative
*Burkholderia thailandensis*	CDC2721121	USA (Louisiana)	Negative
*Burkholderia thailandensis*	CDC2721643	Unknown	Negative
*Burkholderia thailandensis*	CDC2721701	Thailand	Negative
*Burkholderia thailandensis*	CDC2721723	Thailand	Negative
*Burkholderia thailandensis*	CDC2721744	Malaysia	Negative
*Burkholderia humptydooensis*	CDC2721687	Australia	Negative
*Burkholderia oklahomensis*	CDC4002358	USA (Oklahoma)	Negative
*Burkholderia oklahomensis*	CDC4021865	USA (Oklahoma)	Negative
*Burkholderia oklahomensis*	CDC4021866	USA (Oklahoma)	Negative
*Burkholderia vietnamiensis*	CDC2734483	Vietnam	Negative
*Burkholderia pyrrocinia*	ATCC 15958	Unknown	Negative
*Burkholderia caledonica*	CDC8724197	United Kingdom	Negative
*Burkholderia caribensis*	CDC8724200	Martinique	Negative
*Burkholderia ambifaria*	CDC8724201	USA (Wisconsin)	Negative
*Burkholderia anthina*	CDC8724199	USA (Tennessee)	Negative
*Burkholderia cocovenenans*	CDC2734715	Indonesia	Negative
*Burkholderia ferrariae*	CDC8724209	Brazil	Negative
*Burkholderia hydrophila*	CDC2721759	Thailand	Negative
*Burkholderia fungorum*	ATCC BAA-463	Unknown	Negative
*Burkholderia glathei*	CDC2734719	Germany	Negative
*Burkholderia graminis*	CDC2734716	France	Negative
*Burkholderia hospita*	CDC8724207	Belgium	Negative
*Burkholderia kururiensis*	CDC2734717	China	Negative
*Burkholderia nodosa*	CDC8724205	Brazil	Negative
*Burkholderia phenazinium*	ATCC 33666	Unknown	Negative
*Burkholderia phenoliruptrix*	CDC8724203	USA	Negative
*Burkholderia phymatum*	CDC8724208	French Guiana	Negative
*Burkholderia phytofirmans*	CDC8724204	Germany	Negative
*Burkholderia sacchari*	CDC8724202	Brazil	Negative
*Burkholderia silvatlantica*	ATCC BAA-1244	Brazil	Negative
*Burkholderia rhizoxinica*	DSM19002	Germany	Negative
*Burkholderia endofungorum*	DSM19003	Germany	Negative
*Burkholderia gladioli*	CDC3027208	USA (California)	Negative
